# Supplementary material for: The relative influence of agricultural abandonment and semi-natural habitats on parasitoid diversity and community composition
Source: PLoS One. 2024 Aug 20;19(8):e0303656. doi: 10.1371/journal.pone.0303656 (PMC11335124; doi:10.1371/journal.pone.0303656)
Supplement: S1 Table — Habitats: MasC: Mas de Cano, MasI: Mas de San Ignacio, Men: Menejador, VenC: VCarrasqueta, CavB: Caveta del Buitre, FoA: Foia Ampla, MasP: Mas del Parral, Ret: Retura; Trophic guilds: Coc: parasitoids of cocoons, Fun: parasitoid of fungiphage larvae, cPh: parasitoid of concealed phytophages, gPh: grazing phytophages, Mel: parasitoid of melitophages, Pol: poliphage parasitoids, Sap: parasitoid of saprophages, Xyl: parasitoid of xylophages, Unk: hosts unknown, Zoo: parasitoid of zoophages. Strategy: K: koinobionts, I: idiobionts Ec: ectoparasitoids, En: endoparasitoids. (DOCX) [file pone.0303656.s001.docx]

S1 Table

|  | Carrasqueta Mountain | | | | | Mariola  Mountain | | | | | Trophic guild | Strategy |
| --- | --- | --- | --- | --- | --- | --- | --- | --- | --- | --- | --- | --- |
|  | MasC | MasI | Men | VenC | Total | CavB | FoA | MasP | Ret | Total |  |  |
| **ACAENITINAE** |  |  |  |  |  |  |  |  |  |  |  |  |
| *Phaenolobus areolator* (Constantineanu & Constantineanu, 1968) | 2 | 0 | 0 | 0 | 2 | 0 | 0 | 0 | 0 | 0 | Xyl | KEn |
| *Phaenolobus fulvicornis* (Gravenhorst, 1829) | 0 | 0 | 0 | 0 | 0 | 0 | 0 | 1 | 0 | 1 | Xyl | KEn |
| *Phaenolobus nigripennis* (Gravenhorst, 1829) | 0 | 0 | 0 | 0 | 0 | 0 | 2 | 2 | 1 | 5 | Xyl | KEn |
| **ANOMALONINAE** |  |  |  |  |  |  |  |  |  |  |  |  |
| *Agrypon anomelas* (Gravenhorst, 1829) | 0 | 0 | 0 | 4 | 4 | 1 | 0 | 0 | 0 | 1 | gPh | KEn |
| *Agrypon delarvatum* (Gravenhorst, 1829) | 0 | 0 | 2 | 0 | 2 | 1 | 0 | 0 | 0 | 1 | gPh | KEn |
| *Agrypon* sp. 1 | 0 | 0 | 2 | 3 | 5 | 1 | 3 | 0 | 4 | 8 | gPh | KEn |
| *Anomalon cruentatum* (Geoffroy, 1785) | 3 | 0 | 3 | 1 | 7 | 1 | 7 | 12 | 0 | 20 | Sap | KEn |
| *Barylypa* sp. 1 | 0 | 0 | 0 | 0 | 0 | 0 | 0 | 2 | 0 | 2 | gPh | KEn |
| *Barylypa* sp. 2 | 0 | 0 | 0 | 0 | 0 | 0 | 0 | 1 | 0 | 1 | gPh | KEn |
| *Therion cincunflexum* (Linnaeus, 1758) | 0 | 0 | 0 | 0 | 0 | 0 | 1 | 1 | 0 | 2 | gPh | KEn |
| **BANCHINAE** |  |  |  |  |  |  |  |  |  |  |  |  |
| *Cryptopimpla arvicola* (Gravenhorst, 1829) | 0 | 0 | 0 | 0 | 0 | 1 | 5 | 0 | 2 | 8 | gPh | KEn |
| *Cryptopimpla calceolata* (Gravenhrst, 1829) | 1 | 0 | 0 | 0 | 1 | 1 | 0 | 0 | 0 | 1 | gPh | KEn |
| *Exetastes adpressorius* (Thumberg, 1822) | 5 | 0 | 0 | 1 | 6 | 2 | 2 | 0 | 2 | 6 | gPh | KEn |
| *Exetastes calobatus* Gravenhorst, 1829 | 0 | 0 | 0 | 1 | 1 | 5 | 2 | 1 | 0 | 8 | gPh | KEn |
| *Exetastes tibialis* Pfankuch,1921 | 0 | 0 | 0 | 0 | 0 | 7 | 7 | 0 | 7 | 21 | gPh | KEn |
| *Glypta (Glypta) bifoveolata* Gravenhorst, 1829 | 11 | 0 | 1 | 0 | 12 | 0 | 0 | 22 | 0 | 22 | cPh | KEn |
| *Glypta* (*Glypta*) *longispinis* (Gmelin, 1790) | 7 | 1 | 0 | 2 | 10 | 0 | 0 | 1 | 0 | 1 | cPh | KEn |
| *Lissonota bivittata* Gravenhorst, 1829 | 37 | 0 | 6 | 1 | 44 | 6 | 0 | 5 | 0 | 11 | cPh | KEn |
| *Lissonota buccator* (Thumberg, 1822) | 0 | 0 | 0 | 0 | 0 | 1 | 0 | 0 | 0 | 1 | cPh | KEn |
| *Lissonota clypeator* (Gravenhorst, 1820) | 0 | 0 | 0 | 0 | 0 | 0 | 0 | 1 | 0 | 1 | cPh | KEn |
| *Lissonota coracina* (Gmelin, 1790) | 1 | 0 | 0 | 0 | 1 | 0 | 0 | 1 | 0 | 1 | cPh | KEn |
| *Lissonota gracilenta* Holmgren, 1860 | 4 | 5 | 22 | 36 | 67 | 0 | 5 | 2 | 3 | 10 | cPh | KEn |
| *Lissonota impressor* Gravenhorst, 1829 | 15 | 0 | 1 | 0 | 16 | 4 | 1 | 34 | 1 | 40 | cPh | KEn |
| *Lissonota oculatoria* (Fabricius, 1798) | 0 | 0 | 0 | 4 | 4 | 0 | 0 | 1 | 0 | 1 | Pol | KEn |
| *Lissonota picticoxis* Schmiedeknecht, 1900 | 0 | 0 | 1 | 0 | 1 | 0 | 0 | 0 | 0 | 0 | cPh | KEn |
| *Lissonota pimplator* (Zetterstedt, 1838) | 0 | 1 | 0 | 0 | 1 | 1 | 2 | 0 | 0 | 3 | cPh | KEn |
| *Lissonota proxima* Foscolombe, 1854 | 0 | 0 | 0 | 0 | 0 | 2 | 4 | 0 | 0 | 6 | cPh | KEn |
| *Lissonota tenerrima* Thomson, 1877 | 11 | 1 | 5 | 5 | 22 | 0 | 0 | 14 | 0 | 14 | cPh | KEn |
| *Odinophora dorsalis* (Gravenhorst, 1829) | 0 | 7 | 2 | 1 | 10 | 0 | 8 | 7 | 0 | 15 | Unk | KEn |
| *Syzeuctus fuscator* (Panzer, 1809) | 0 | 6 | 7 | 25 | 38 | 2 | 11 | 9 | 2 | 24 | gPh | KEn |
| *Syzeuctus inaequalis* (Foscolombe, 1854) | 0 | 6 | 21 | 9 | 36 | 1 | 0 | 1 | 0 | 2 | gPh | KEn |
| *Syzeuctus petiolaris* (Gravenhorst, 1829) | 1 | 0 | 0 | 0 | 1 | 0 | 2 | 0 | 0 | 2 | gPh | KEn |
| *Syzeuctus puberulus* (Kriechbaumer, 1895) | 0 | 0 | 0 | 0 | 0 | 0 | 3 | 0 | 0 | 3 | gPh | KEn |
| *Syzeuctus tigris* Seyrig, 1926 | 0 | 0 | 0 | 0 | 0 | 0 | 1 | 0 | 0 | 1 | gPh | KEn |
| **COLLYRIINAE** |  |  |  |  |  |  |  |  |  |  |  |  |
| *Collyria coxator* (Villers, 1789) | 4 | 0 | 2 | 0 | 6 | 0 | 0 | 0 | 0 | 0 | cPh | KEn |
| *Collyria disticnta* Izquierdo & Rey del Castillo, 1985 | 1 | 1 | 0 | 0 | 2 | 150 | 1 | 0 | 2 | 153 | cPh | KEn |
| *Collyria iberica* Schmiedeknecht, 1908 | 28 | 10 | 15 | 2 | 55 | 0 | 0 | 6 | 0 | 6 | cPh | KEn |
| **CREMASTINAE** |  |  |  |  |  |  |  |  |  |  |  |  |
| *Cremastus aegyptiacus* Szépligeti, 1905 | 0 | 1 | 0 | 1 | 2 | 0 | 1 | 3 | 1 | 5 | cPh | KEn |
| *Cremastus lineatus ibericus* Kolarov, 1996 | 1 | 0 | 0 | 0 | 1 | 1 | 0 | 1 | 5 | 7 | cPh | KEn |
| *Cremastus puberulus*  Szépligeti, 1899 | 0 | 0 | 0 | 3 | 3 | 1 | 5 | 1 | 0 | 7 | cPh | KEn |
| *Cremastus spectator* Gravenhorst, 1829 | 0 | 0 | 1 | 0 | 1 | 0 | 0 | 0 | 0 | 9 | cPh | KEn |
| *Cremastus* sp. 1 | 0 | 0 | 2 | 2 | 4 | 0 | 0 | 0 | 0 | 0 | cPh | KEn |
| *Cremastus* sp. 2 | 0 | 1 | 0 | 0 | 1 | 0 | 0 | 0 | 0 | 0 | cPh | KEn |
| *Eucremastus manni* (Tschek, 1871) | 0 | 1 | 0 | 0 | 1 | 0 | 1 | 0 | 0 | 1 | cPh | KEn |
| *Pristomerus armatus* (Lucas, 1849) | 0 | 0 | 0 | 0 | 0 | 0 | 1 | 1 | 0 | 2 | cPh | KEn |
| *Pristomerus kasparyani* Narolsky, 1986 | 0 | 0 | 0 | 0 | 0 | 0 | 0 | 1 | 0 | 1 | cPh | KEn |
| *Pristomerus luridus* Kokujev, 1905 | 1 | 0 | 5 | 3 | 9 | 2 | 4 | 5 | 1 | 12 | cPh | KEn |
| *Pristomerus mesopotamicus* Horstmann, 1990 | 0 | 0 | 0 | 0 | 0 | 0 | 0 | 1 | 0 | 1 | cPh | KEn |
| *Temelucha decorata* (Gravenhorst, 1829) | 11 | 0 | 0 | 3 | 14 | 0 | 11 | 13 | 1 | 25 | cPh | KEn |
| *Temelucha genalis* (Szepligeti, 1899) | 0 | 0 | 0 | 0 | 0 | 0 | 2 | 18 | 0 | 38 | cPh | KEn |
| *Temelucha interruptor* (Gravehorst, 1829) | 4 | 0 | 0 | 3 | 7 | 0 | 12 | 2 | 0 | 14 | cPh | KEn |
| *Temelucha longicauda* Kolarov, 1996 | 0 | 0 | 0 | 0 | 0 | 0 | 0 | 2 | 0 | 2 | cPh | KEn |
| *Temelucha pseudocaudata* Kolarov, 1982 | 0 | 0 | 0 | 1 | 1 | 0 | 0 | 0 | 0 | 0 | cPh | KEn |
| *Temelucha tricolorata* Sedivy, 1968 | 1 | 0 | 0 | 0 | 1 | 0 | 0 | 0 | 0 | 0 | cPh | KEn |
| *Temelucha variipes* (Szépligeti, 1899) | 5 | 0 | 0 | 0 | 5 | 0 | 0 | 1 | 0 | 1 | cPh | KEn |
| **CRYPTINAE** |  |  |  |  |  |  |  |  |  |  |  |  |
| **Cryptini** |  |  |  |  |  |  |  |  |  |  |  |  |
| *Agrothereutes australis* (Habermehl, 1926) | 0 | 0 | 0 | 1 | 1 | 0 | 0 | 0 | 0 | 0 | gPh | IEc |
| *Agrothereutes parvulus* (Habermehl, 1926) | 2 | 0 | 1 | 0 | 3 | 4 | 0 | 0 | 1 | 5 | gPh | IEc |
| *Agrothereutes tunetanus* (Habermehl, 1925) | 0 | 0 | 0 | 2 | 2 | 0 | 0 | 0 | 0 | 0 | gPh | IEc |
| *Aritranis claviventris* (Kriechbaumer, 1894) | 1 | 0 | 0 | 0 | 1 | 0 | 0 | 1 | 0 | 1 | gPh | IEc |
| *Aritranis director* (Thunberg, 1822) | 6 | 1 | 5 | 2 | 14 | 33 | 12 | 1 | 2 | 48 | gPh | IEc |
| *Aritranis longicauda* (Kriechbaumer, 1873) | 36 | 2 | 0 | 10 | 48 | 11 | 48 | 2 | 4 | 65 | gPh | IEc |
| *Aritranis occisor* (Gravenhorst, 1829) | 7 | 0 | 1 | 4 | 12 | 14 | 22 | 4 | 32 | 72 | gPh | IEc |
| *Buathra tarsoleucos* (Schrank, 1781) | 3 | 0 | 0 | 0 | 3 | 0 | 0 | 0 | 0 | 0 | gPh | IEc |
| *Cryptus speciosus* Tosquinet, 1896 | 1 | 0 | 0 | 0 | 1 | 1 | 0 | 2 | 1 | 4 | gPh | IEc |
| *Cryptus bucculentus* Tschek, 1871 | 1 | 1 | 0 | 2 | 4 | 1 | 1 | 0 | 0 | 2 | gPh | IEc |
| *Cryptus moschator* (Fabricius, 1787) | 0 | 0 | 2 | 0 | 2 | 0 | 0 | 0 | 0 | 0 | gPh | IEc |
| *Cryptus spinosus* Gravenhorst, 1829 | 2 | 0 | 2 | 4 | 8 | 2 | 1 | 2 | 0 | 5 | gPh | IEc |
| *Cryptus spiralis* (Geoffroy, 1785) | 0 | 0 | 0 | 0 | 0 | 0 | 1 | 1 | 0 | 2 | gPh | IEc |
| *Cryptus tuberculatus* Gravenhorst, 1829 | 0 | 0 | 1 | 0 | 1 | 0 | 0 | 0 | 0 | 0 | gPh | IEc |
| *Cryptus* sp. 1 | 0 | 0 | 0 | 0 | 0 | 0 | 3 | 1 | 0 | 4 | gPh | IEc |
| *Enclisis castellana* Bordera, Kolarov & Mazón, 2008 | 0 | 0 | 0 | 1 | 1 | 0 | 0 | 0 | 0 | 0 | Xyl | IEc |
| *Enclisis infernator (*Aubert, 1968) | 0 | 0 | 0 | 0 | 0 | 18 | 0 | 0 | 0 | 18 | Xyl | IEc |
| *Enclisis ornaticeps* (Thomson, 1885) | 0 | 0 | 0 | 1 | 1 | 0 | 0 | 0 | 0 | 0 | Xyl | IEc |
| *Enclisis schwarzi* Bordera & Hernández-Rodríguez, 2003 | 1 | 0 | 2 | 3 | 6 | 89 | 0 | 0 | 5 | 94 | Xyl | IEc |
| *Enclisis vindex* (Tschek, 1871) | 0 | 0 | 0 | 6 | 6 | 0 | 1 | 0 | 1 | 2 | Xyl | IEc |
| *Hoplocryptus bellosus* (Curtis, 1837) | 0 | 0 | 0 | 0 | 0 | 1 | 0 | 0 | 0 | 1 | Mel | IEc |
| *Hoplocryptus femoralis* (Gravenhorst, 1829) | 0 | 0 | 0 | 0 | 0 | 0 | 1 | 0 | 0 | 1 | Mel | IEc |
| *Hoplocryptus heliophilus* (Tschek, 1871) | 30 | 4 | 1 | 9 | 44 | 7 | 13 | 17 | 9 | 46 | Mel | IEc |
| *Hoplocryptus melanocephalus* (Gravenhorst, 1829) | 12 | 0 | 0 | 0 | 12 | 15 | 1 | 4 | 12 | 32 | Mel | IEc |
| *Hoplocryptus murarius* (Börner, 1782) | 0 | 0 | 0 | 0 | 0 | 0 | 5 | 0 | 1 | 6 | Mel | IEc |
| *Hoplocryptus odoriferator* (Dufour & Perris, 1840) | 7 | 0 | 0 | 0 | 7 | 4 | 2 | 0 | 2 | 8 | Mel | IEc |
| *Hoplocryptus quadriguttatus* (Gravenhorst, 1829) | 0 | 0 | 0 | 0 | 0 | 0 | 0 | 0 | 1 | 1 | Mel | IEc |
| *Ischnus agitator* (Olivier, 1792) | 1 | 0 | 0 | 0 | 1 | 1 | 1 | 1 | 0 | 3 | gPh | IEc |
| *Latibulus lautus* (Tosquinet, 1896) | 0 | 0 | 0 | 0 | 0 | 0 | 0 | 1 | 1 | 2 | Zoo | IEc |
| *Listrognathus firmator* (Fabricius, 1798) | 0 | 0 | 0 | 0 | 0 | 0 | 1 | 0 | 0 | 1 | gPh | IEc |
| *Listrognathus pubescens* (Fonscolombe, 1850) | 1 | 0 | 0 | 0 | 1 | 0 | 2 | 0 | 1 | 3 | gPh | IEc |
| *Meringopus nigerrimus* (Fonscolombe, 1850) | 0 | 0 | 0 | 1 | 1 | 0 | 0 | 0 | 1 | 1 | gPh | IEc |
| *Meringopus pseudonymus* (Tschek, 1872) | 21 | 0 | 0 | 0 | 21 | 1 | 2 | 1 | 0 | 4 | gPh | IEc |
| *Meringopus titillator* (Linnaeus, 1758) | 0 | 0 | 0 | 0 | 0 | 0 | 0 | 2 | 0 | 2 | gPh | IEc |
| *Meringopus turanus* (Habermehl, 1918) | 0 | 0 | 0 | 0 | 0 | 0 | 0 | 1 | 0 | 1 | gPh | IEc |
| *Mesostenus albinotatus* Gravenhorst, 1829 | 0 | 22 | 8 | 127 | 157 | 19 | 10 | 7 | 37 | 73 | gPh | IEc |
| *Mesostenus dentifer* Thomson, 1896 | 1 | 0 | 0 | 0 | 1 | 0 | 2 | 0 | 1 | 3 | gPh | IEc |
| *Mesostenus grammicus* Gravenhorst, 1829 | 0 | 2 | 0 | 25 | 27 | 0 | 1 | 0 | 5 | 6 | gPh | IEc |
| *Mesostenus transfuga* Gravenhorst, 1829 | 1 | 0 | 1 | 3 | 5 | 2 | 1 | 0 | 0 | 3 | gPh | IEc |
| *Mesostenus* sp. 1 | 0 | 2 | 0 | 9 | 11 | 0 | 0 | 0 | 4 | 4 | gPh | IEc |
| *Myrmeleonostenus italicus* (Gravenhorst, 1829) | 0 | 0 | 0 | 0 | 0 | 0 | 0 | 0 | 1 | 1 | Zoo | IEc |
| *Synechocryptus mactator* (Tschek, 1871) | 4 | 1 | 1 | 0 | 6 | 0 | 0 | 0 | 0 | 0 | gPh | IEc |
| *Trychosis legator* (Thunberg, 1822) | 16 | 18 | 8 | 24 | 66 | 15 | 72 | 40 | 23 | 150 | Pol | IEc |
| *Trychosis neglecta* (Tschek, 1871) | 0 | 0 | 0 | 0 | 0 | 0 | 1 | 0 | 0 | 1 | Pol | IEc |
| *Xylophrurus augustus* (Dalman, 1823) | 7 | 0 | 1 | 8 | 16 | 2 | 1 | 2 | 4 | 9 | Pol | IEc |
| *Xylophrurus* sp. 1 | 12 | 30 | 12 | 39 | 93 | 14 | 8 | 3 | 13 | 38 | Unk | IEc |
| *Xylophrurus* sp. 2 | 0 | 0 | 1 | 0 | 1 | 0 | 0 | 0 | 0 | 0 | Unk | IEc |
| **DIPLAZONTINAE** |  |  |  |  |  |  |  |  |  |  |  |  |
| *Diplazon laetatorius* (Fabricius, 1781) | 3 | 0 | 0 | 0 | 3 | 2 | 3 | 14 | 1 | 20 | Zoo | KEn |
| *Syrphoctonus nigritarsus* (Gravenhorst, 1829) | 0 | 0 | 0 | 0 | 0 | 0 | 1 | 0 | 0 | 1 | Zoo | KEn |
| *Syrphophilus bizonarius* (Gravenhorst, 1829) | 1 | 0 | 0 | 0 | 1 | 0 | 0 | 4 | 0 | 4 | Zoo | KEn |
| **METOPIINAE** |  |  |  |  |  |  |  |  |  |  |  |  |
| *Trieces confusus* Mazón & Bordera, 2020 | 0 | 0 | 2 | 0 | 2 | 0 | 0 | 0 | 0 | 0 | cPh | KEn |
| *Exochus coronatus* Gravenhorst, 1829 | 1 | 0 | 0 | 0 | 1 | 1 | 0 | 4 | 8 | 13 | cPh | KEn |
| *Exochus mitratus* Gravenhorst, 1829 | 8 | 3 | 8 | 51 | 70 | 8 | 8 | 7 | 6 | 29 | cPh | KEn |
| *Hypsicera femoralis* (Geoffroy, 1785) | 0 | 0 | 0 | 0 | 0 | 0 | 0 | 11 | 1 | 12 | cPh | KEn |
| **OPHIONINAE** |  |  |  |  |  |  |  |  |  |  |  |  |
| *Enicospilus inflexus* (Ratzeburg, 1844) | 0 | 0 | 0 | 1 | 1 | 0 | 0 | 0 | 0 | 0 | gPh | KEn |
| *Enicospilus ramidulus* (Linnaeus, 1758) | 1 | 0 | 0 | 0 | 1 | 0 | 0 | 1 | 0 | 1 | gPh | KEn |
| *Enicospilus merdarius* (Gravenhorst, 1829) | 0 | 1 | 0 | 2 | 3 | 0 | 0 | 0 | 0 | 0 | gPh | KEn |
| *Hellwigiella dichromoptera* (Costa, 1886) | 0 | 0 | 1 | 0 | 1 | 0 | 0 | 0 | 0 | 0 | Unk | KEn |
| *Ophion mocsaryi* Brauns, 1889 | 0 | 0 | 0 | 1 | 1 | 1 | 1 | 0 | 1 | 3 | gPh | KEn |
| *Ophion obscuratus* Fabricius, 1798 | 0 | 0 | 0 | 1 | 1 | 1 | 0 | 0 | 1 | 2 | gPh | KEn |
| *Ophion* sp. 1 | 0 | 0 | 0 | 2 | 2 | 0 | 0 | 0 | 0 | 0 | gPh | KEn |
| **ORTHOCENTRINAE** |  |  |  |  |  |  |  |  |  |  |  |  |
| *Dialipsis exilis* Förster, 1871 | 0 | 0 | 0 | 1 | 1 | 0 | 0 | 0 | 0 | 0 | Fun | KEn |
| *Megastylus flavopictus* (Gravenhorst, 1829) | 0 | 0 | 0 | 0 | 0 | 0 | 0 | 0 | 1 | 1 | Unk | KEn |
| *Megastylus impressor* Schiødte, 1838 | 0 | 0 | 0 | 0 | 0 | 2 | 0 | 0 | 0 | 2 | Zoo | KEn |
| *Orthocentrus asper* (Gravenhorst, 1829) | 0 | 0 | 0 | 1 | 1 | 3 | 0 | 0 | 3 | 6 | Fun | KEn |
| *Orthocentrus orbitator* Aubert, 1963 | 0 | 0 | 0 | 0 | 0 | 1 | 0 | 0 | 1 | 2 | Unk | KEn |
| *Orthocentrus protervus* Holmgren, 1858 | 0 | 0 | 0 | 0 | 0 | 0 | 0 | 0 | 1 | 1 | Fun | KEn |
| *Orthocentrus winnertzii* Förster, 1850 | 0 | 0 | 0 | 0 | 0 | 1 | 0 | 0 | 1 | 2 | Pol | KEn |
| *Picrostigeus recticauda* (Thomson, 1897) | 0 | 0 | 0 | 0 | 0 | 1 | 0 | 0 | 0 | 1 | Unk | KEn |
| *Plectiscus* sp. 1 | 0 | 0 | 0 | 2 | 2 | 0 | 0 | 0 | 0 | 0 | Unk | KEn |
| *Stenomacrus affinitor* Aubert, 1981 | 0 | 0 | 0 | 2 | 2 | 3 | 1 | 0 | 0 | 4 | cPh | KEn |
| *Stenomacrus caudatus* (Holmgren, 1858) | 0 | 0 | 0 | 1 | 1 | 0 | 0 | 0 | 0 | 0 | Unk | KEn |
| *Stenomacrus* sp. 1 | 0 | 0 | 0 | 0 | 0 | 2 | 0 | 0 | 0 | 2 | Unk | KEn |
| *Symplecis invisitata* Rossem, 1981 | 1 | 0 | 1 | 5 | 7 | 0 | 0 | 0 | 10 | 10 | Fun | KEn |
| **ORTHOPELMATINAE** |  |  |  |  |  |  |  |  |  |  |  |  |
| *Orthopelma brevicorne* Morley, 1907 | 0 | 0 | 1 | 0 | 1 | 0 | 0 | 1 | 0 | 1 | cPh | KEn |
| **PIMPLINAE** |  |  |  |  |  |  |  |  |  |  |  |  |
| **Ephialtini** |  |  |  |  |  |  |  |  |  |  |  |  |
| *Clistopyga incitator* (Fabricius, 1793) | 1 | 0 | 0 | 11 | 12 | 4 | 6 | 4 | 6 | 20 | Zoo | IEc |
| *Dolichomitus kriechbaumeri* (Schulz, 1906) | 0 | 0 | 0 | 0 | 0 | 1 | 0 | 0 | 0 | 1 | Xyl | IEc |
| *Endromopoda detrita* (Holmgren, 1860) | 2 | 0 | 1 | 0 | 3 | 0 | 0 | 0 | 0 | 0 | Pol | IEc |
| *Exeristes roborator* (Fabricius, 1793) | 7 | 1 | 1 | 1 | 10 | 23 | 5 | 6 | 0 | 34 | Pol | IEc |
| *Pseudopimpla propodeumpunctata* Mazón & Bordera, 2010 | 0 | 2 | 0 | 0 | 2 | 0 | 0 | 0 | 0 | 0 | Xyl | IEc |
| *Zatypota bohemani* (Holmgren, 1860) | 0 | 0 | 0 | 0 | 0 | 0 | 0 | 1 | 0 | 1 | Zoo | KEc |
| **Pimplini** |  |  |  |  |  |  |  |  |  |  |  |  |
| *Itoplectis maculator* (Fabricius, 1775) | 1 | 0 | 0 | 0 | 1 | 0 | 0 | 1 | 0 | 1 | Coc | IEn |
| *Pimpla rufipes* (Miller, 1759) | 0 | 0 | 0 | 0 | 0 | 0 | 0 | 1 | 0 | 1 | Coc | IEn |
| *Pimpla spuria* Gravenhorst, 1829 | 12 | 4 | 0 | 4 | 20 | 29 | 9 | 10 | 0 | 48 | Coc | IEn |
| **TERSILOCHINAE** |  |  |  |  |  |  |  |  |  |  |  |  |
| *Aneuclis incidens* (Thomson, 1889) | 0 | 0 | 0 | 0 | 0 | 2 | 1 | 1 | 0 | 4 | Mel | KEn |
| *Barycnemis* sp. 1 | 0 | 0 | 0 | 1 | 1 | 0 | 0 | 0 | 16 | 16 | cPh | KEn |
| *Diaparsis* (*Diaparsis*) *temporalis* Horstmann, 1979 | 0 | 0 | 2 | 0 | 2 | 0 | 0 | 0 | 0 | 0 | gPh | KEn |
| *Diaparsis* (*Nanodiaparsis*) *aperta* (Thomson, 1889) | 0 | 0 | 1 | 0 | 1 | 0 | 0 | 0 | 1 | 1 | Xyl | KEn |
| *Diaparsis* sp. 1 | 0 | 0 | 0 | 0 | 0 | 1 | 0 | 0 | 0 | 1 | Unk | KEn |
| *Phradis corsicator* (Aubert, 1969) | 2 | 0 | 0 | 0 | 2 | 1 | 0 | 0 | 0 | 1 | Mel | KEn |
| *Phradis mediterraneus* Khalaim, Bordera & Rodríguez-Berrío, 2009 | 0 | 1 | 1 | 1 | 3 | 3 | 0 | 0 | 0 | 3 | Mel | KEn |
| *Phradis minutus* (Bridgman, 1889) | 0 | 0 | 0 | 1 | 1 | 0 | 0 | 0 | 0 | 0 | Mel | KEn |
| *Sathropterus pumilus* (Holmgren, 1860) | 0 | 0 | 0 | 0 | 0 | 0 | 0 | 1 | 0 | 1 | Unk | KEn |
| *Tersilochus* (*Gonolochus*) *stenocari* (Gregor, 1941) | 0 | 0 | 0 | 0 | 0 | 0 | 0 | 1 | 0 | 1 | Xyl | KEn |
| *Tersilochus* (*Tersilochus*) *obscurator* (Aubert, 1959) | 0 | 0 | 2 | 0 | 2 | 0 | 0 | 0 | 0 | 0 | cPh | KEn |
| *Tersilochus* (*Tersilochus*) *subdepressus* Thomson, 1889 | 0 | 0 | 0 | 1 | 1 | 0 | 0 | 0 | 0 | 0 | Unk | KEn |
| **TRYPHONINAE** |  |  |  |  |  |  |  |  |  |  |  |  |
| **Exenterini** |  |  |  |  |  |  |  |  |  |  |  |  |
| *Cycasis rubiginosa* (Gravenhorst, 1829) | 1 | 0 | 5 | 0 | 6 | 3 | 0 | 2 | 3 | 8 | gPh | KEc |
| **Phytodietini** |  |  |  |  |  |  |  |  |  |  |  |  |
| *Netelia* (*Netelia*) *denticulator* Aubert, 1969 | 0 | 1 | 0 | 0 | 1 | 0 | 0 | 0 | 0 | 0 | gPh | KEc |
| *Netelia* (*Netelia*) *fuscicornis* Holmgren, 1860 | 1 | 0 | 0 | 0 | 1 | 0 | 0 | 0 | 2 | 2 | gPh | KEc |
| *Netelia* (*Netelia*) *testacea* (Gravenhorst, 1829) | 1 | 0 | 0 | 0 | 1 | 1 | 1 | 7 | 2 | 11 | gPh | KEc |
| *Netelia* (*Paropheltes*) *ornata* (Vollenhoven, 1873) | 0 | 0 | 0 | 1 | 1 | 0 | 0 | 0 | 0 | 0 | gPh | KEc |
| **Tryphonini** |  |  |  |  |  |  |  |  |  |  |  |  |
| *Tryphon* (*Tryphon*) *atriceps* Stephens, 1835 | 19 | 0 | 0 | 0 | 19 | 0 | 0 | 11 | 0 | 11 | gPh | KEc |
| *Tryphon* (*Tryphon*) *rutilator* (Linnaeus, 1761) | 24 | 0 | 2 | 1 | 27 | 0 | 0 | 0 | 0 | 0 | gPh | KEc |
| *Tryphon* (*Tryphon*) *signator* Gravenhorst, 1829 | 0 | 0 | 0 | 0 | 0 | 1 | 0 | 0 | 0 | 1 | gPh | KEc |
| **XORIDINAE** |  |  |  |  |  |  |  |  |  |  |  |  |
| *Xorides* (*Gonophonus*) *propinquus* (Tschek, 1869) | 0 | 4 | 0 | 14 | 18 | 0 | 1 | 0 | 0 | 1 | Xyl | IEc |
| *Xorides* (*Xorides*) *gravenhorstii* (Curtis, 1831) | 0 | 0 | 0 | 0 | 0 | 0 | 0 | 1 | 0 | 1 | Xyl | IEc |
