# Supplementary material for: The relative influence of agricultural abandonment and semi-natural habitats on parasitoid diversity and community composition
Source: PLoS One. 2024 Aug 20;19(8):e0303656. doi: 10.1371/journal.pone.0303656 (PMC11335124; doi:10.1371/journal.pone.0303656)
Supplement: S2 Table — (DOCX) [file pone.0303656.s002.docx]

S2 Table

| Response | Buffer | Time since | Proportion of |
| --- | --- | --- | --- |
| variable | radii | agr. abandonement | forested landscape |
|  |  | χ21,6 (p-value) | χ21,6 (p-value) |
| Taxonomic richness | 100 | 0.01 (0.9173) | 2.37 (0.1234) |
| Taxonomic evenness | 100 | 0.11 (0.7451) | **4.29 (0.0384)** |
| LCBD | 100 | 2.25 (0.1339) | **19.25 (<0.0001)** |
| Total abundance | 100 | 0.21 (0.6463) | 0.34 (0.5572) |
| Guild richness | 100 | 0.83 (0.3617) | 0.55 (0.4602) |
| Guild evenness | 100 | <0.01 (0.9627) | 0.42 (0.5186) |
| Taxonomic richness | 200 | <0.01 (0.9980) | 2.09 (0.1479) |
| Taxonomic evenness | 200 | 0.86 (0.3551) | **9.96 (0.0016)** |
| LCBD | 200 | 3.74 (0.0530) | **31.15 (<0.0001)** |
| Total abundance | 200 | 0.23 (0.6321) | 0.31 (0.5794) |
| Guild richness | 200 | 0.38 (0.5368) | 0.95 (0.3291) |
| Guild evenness | 200 | 0.02 (0.8978) | 0.61 (0.434) |
| Taxonomic richness | 500 | 0.12 (0.7269) | 0.48 (0.49) |
| Taxonomic evenness | 500 | 0.29 (0.5876) | **5.22 (0.0224)** |
| LCBD | 500 | 2.85 (0.0915) | **20.47 (<0.0001)** |
| Total abundance | 500 | 0.34 (0.5607) | 0.59 (0.4419) |
| Guild richness | 500 | 0.16 (0.6910) | 3.04 (0.0813) |
| Guild evenness | 500 | <0.01 (0.9641) | 0.28 (0.5947) |
| Taxonomic richness | 1000 | 0.57 (0.4505) | 0.01 (0.9284) |
| Taxonomic evenness | 1000 | <0.01 (0.9985) | 1.15 (0.2832) |
| LCBD | 1000 | 1.49 (0.2225) | 3.77 (0.0523) |
| Total abundance | 1000 | 0.70 (0.4031) | 1.5 (0.2208) |
| Guild richness | 1000 | 0.12 (0.7245) | 2.92 (0.0874) |
| Guild evenness | 1000 | 0.05 (0.8297) | 1.08 (0.2992) |
| Taxonomic richness | 1500 | 0.81 (0.3668) | 0.23 (0.6299) |
| Taxonomic evenness | 1500 | 0.19 (0.6632) | 0.22 (0.6373) |
| LCBD | 1500 | 0.57 (0.45) | 0.57 (0.4515) |
| Total abundance | 1500 | 0.15 (0.703) | 0.20 (0.6554) |
| Guild richness | 1500 | 1.06 (0.3038) | 0.32 (0.5717) |
| Guild evenness | 1500 | <0.01 (0.9674) | 0.47 (0.4907) |
| Taxonomic richness | 2000 | 0.49 (0.4847) | 0.07 (0.7882) |
| Taxonomic evenness | 2000 | 0.26 (0.6098) | 0.49 (0.4845) |
| LCBD | 2000 | 0.53 (0.4655) | 1.19 (0.2748) |
| Total abundance | 2000 | 0.07 (0.7877) | 0.05 (0.8200) |
| Guild richness | 2000 | 1.86 (0.1722) | <0.01 (0.9771) |
| Guild evenness | 2000 | 0.01 (0.9155) | 0.02 (0.8818) |
