# Supplementary material for: The relative influence of agricultural abandonment and semi-natural habitats on parasitoid diversity and community composition
Source: PLoS One. 2024 Aug 20;19(8):e0303656. doi: 10.1371/journal.pone.0303656 (PMC11335124; doi:10.1371/journal.pone.0303656)
Supplement: Attachment — Submitted filename: Reply to reviewers.pdf [file pone.0303656.s003.pdf]

Centre de Biologie pour la Gestion des Populations - CBGP
Campus International de Baillarguet
755 avenue du Campus Agropolis - CS 30016
34988 Montferrier-sur-Lez - France

Reply to reviewer #1

The paper addresses an important subject about the influence of the time of agricultural abandonment and proportion of semi-natural habitats on taxonomic and functional diversity of parasitoids. It is an interesting topic and the text is clear and easy to follow. My main concerns are the number of sampled sites that are very few and some issues in introduction and discussion.

We agree with the reviewer that one of the limitations of our study is the limited number of sites studied. We discuss this limitation below and in our reply to reviewer #2.

Particularly, the mechanisms for which time of agricultural abandonment (local scale) could affect parasitoids are not explicitly explained. For example, could one driver be the increasing plant richness and biomass with increasing time of agricultural abandonment? I understand the authors did not measure any of the variables related to plants and herbivores, but I consider that more explanations of how lower trophic levels can change with the time of abandonment are needed.

We have included a more mechanistic explanation (including the effect of plant diversity) to the biodiversity-biocontrol relationship in the introductory section.

Another important point is about this explanatory variable which is considered and analyzed by the authors as a continuous variable of different times of abandonment, however it involves only a few levels of time: not abandoned (active croplands), recently abandoned (10 and 20 years ago abandoned crops) and wild sites (considered as 75 years ago abandoned crops) and these look like different stages of succession of sites.

We agree with the reviewer that considering time since agricultural abandonment as a categorical variable would be a potential way to analyse our data. We prefer, however, to keep this variable as continuous for two reasons. The first and main reason is conceptual. Our study aims at exploring how time since agricultural abandonment affects parasitoid communities, and not how different succession states alter these communities. The second reason is based on the fact that by converting this variable into a continuous one we gain statistical power. We studied several sites comprising four different times since

Centre de Biologie pour la Gestion des Populations - CBGP
Campus International de Baillarguet
755 avenue du Campus Agropolis - CS 30016
34988 Montferrier-sur-Lez - France

agricultural abandonment. By considering this variable as continuous we lose one degree of freedom, but we lose three if the variable is considered as categorical. Considering that an important limitation of our study is the limited number of sites studied (as pointed by Reviewer #2 - see our reply to this question below) we believe this is a good choice. We agree with the reviewer that these two issues were not clear enough in our manuscript and we have explained them clearly in this new version.

Specific points
Introduction
- 4th paragraph. "the relative contribution of two different scales to alpha and beta", do authors mean spatial scales?

We meant different levels of influence, so we have replaced "scales" by "layers".

- Last paragraph. Points 1 to 4 are confusing because I do not understand if they are a list of the hypothesis or predictions and/or questions to be answered.

The hypothesis section has been fully rewritten (see comment to Reviewer #2).

Methods
- Paragraph of study area. It would be useful to mention here the most dominant plant species in semi-natural habitats.

As already mentioned in the previous version these habitats were dominated by *Quercus ilex* subsp. *rotundifolia* and *Q. coccifera.*

- It is not clear the explanation about selection and number of sites. It seems that authors sampled 12 sites in total (6 in each mountain) according to the written "In each mountain range, we selected four sites along a gradient of time since agriculture was abandoned, from a wild protected area to a managed cropland, and two sites at different stages of succession (i.e. ten and twenty years from agricultural abandonment). And also, it is confusing why only two sites are in different stages of succession.

This paragraph has now been clarified.

Centre de Biologie pour la Gestion des Populations - CBGP
Campus International de Baillarguet
755 avenue du Campus Agropolis - CS 30016
34988 Montferrier-sur-Lez - France

Reply to reviewer #2

This is a very well written paper on the variation in diversity of parasitoid wasps amongst landscape composition. Unfortunately, I believe there are several caveats to this study that prevent the authors from achieving their goals and answering their scientific questions.

First, the authors start with a very nice and throughout background section in their introduction, which is sadly followed by unclear hypotheses statements. For instance, just the first hypothesis could be partitioned into 4 different ones! Even more, the 2nd and 4th hypothesis seem to mix literature background that has not been introduced previously, making them quite hard to fully understand at this stage of the paper! I would suggest the authors to make a comprehensive figure, with graphs showing the expected directionality of each hypothesis, I believe it would make the text much clearer that way.

We agree with the reviewer. We have fixed these issues at different levels. First, we have included a full new paragraph in the introduction section explaining well beta diversity partitioning and how these metrics can be used to infer parasitoid spillover. We have rephrased the hypothesis section and made it clearer together with a figure showing the hypotheses tested. We have concentrated our hypotheses in three axes (both in the figure and in the text): effects on alpha diversity, effects on beta diversity and effects on functional diversity. We have removed the last hypothesis (and associated results) exploring effects on the different parasitoid guilds.

Second, and this is perhaps my biggest comment, the number of sites that the authors have chosen is not enough to test the hypotheses they put forward. In table 1, the authors present a brief description of their site, in which we can see that there are only 2 replicates per group. The authors briefly acknowledge this is the discussion, but, to me, it severely hinders their capacity to make successful statistical analysis! The major problem in here is that the sites are not spatially isolated (15km of distance between the 2 sites), which means that the sites could be considered as pseudo-replicates at the spatial extent at which the authors work. For instance, considering the larger buffer size at 2000m around the sites for each site would leave only 11km between the 2!

We agree with the reviewer that the main limitation of our study is the small number of sites studied. A limitation that we partially solve, firstly by being very cautious with our conclusions. As this reviewer points out, this

Centre de Biologie pour la Gestion des Populations - CBGP
Campus International de Baillarguet
755 avenue du Campus Agropolis - CS 30016
34988 Montferrier-sur-Lez - France

cautious approach was already included in our previous section, and in this new version we have made an effort to implement it even further. In addition, we increase our statistical power by transforming the variable "time since agricultural abandonment" as continuous (see a related comment to our reply to Reviewer #1). This approach implies that we are not really comparing categories with 2 replicates but a continuous variable with multiple values.

We agree with the reviewer that the 2000m radius is in some cases too large to be considered as independent from nearby radii. In this new version we have considered this limitation as a criteria to select the 500m radii after the multi-scale analysis. In addition we believe that the use of this radii size does not imply pseudo-replication because in the study area landscapes are highly fragmented so landscape changes occur at very short distances. Also, our experimental design whereby the four different site types (in terms of time since agricultural abandonment) are located in two independent mountain ranges (or blocks), increases the independence of the different sites.

We have further ensured that there is no spatial autocorrelation among landscape measures with novel analyses that we have included in this new version. We have used Mantel tests to explore the association between kilometric distances between sites, and differences in the proportion of agricultural lands between sites. For all the different buffer sizes used, the proportion of agricultural land does not relate with the kilometric distance between sites, a result that shows little spatial dependency on the landscape variable used. See the manuscript for further details on these analyses and their results.

Also, as a more minor comment, the statistical analysis section in the method is not clear and needs to be reworded. There are a lot of information in there that are mixed with other information, such as the last sentence of the section. There is also some information missing in the model building, such as Distribution family for each response variables. Please provide the data as inputted in R + R code to make it clear for the reader.

We have made this section clearer and changed the titles of the different subsections. We now provide the R code used to analyse our data, which can be found in a public repository (see text for details).

The result section also needs a lot of work and rewording. I do not understand the S value in the result section. Does it refer to the effect size of your models? If so, please provide 95% CI for each predictor. P-Values do not present any

Centre de Biologie pour la Gestion des Populations

Centre de Biologie pour la Gestion des Populations - CBGP
Campus International de Baillarguet
755 avenue du Campus Agropolis - CS 30016
34988 Montferrier-sur-Lez - France

useful information (Table 2). As I stated above, please present Effect size of each predictor + 95% CI instead.

The S value is the statistic for the non-parametric Spearman's correlation test used to explore the collinearity between time since agricultural abandonment and the proportion of agricultural landscape in each study site. We agree that effect sizes are needed for clarification and are now included in this new version. We prefer to keep p-values instead of CI as a standard measure of fidelity towards our proposed hypotheses.

Minor comments:
Methods:
I do not understand why you gave numerical values to your sites, and it is not clear how you performed the analysis.

See our comment to reviewer #1

Make your pace of sampling clearer in the methods

Done
