# Supplementary material for: The relative influence of agricultural abandonment and semi-natural habitats on parasitoid diversity and community composition
Source: PLoS One. 2024 Aug 20;19(8):e0303656. doi: 10.1371/journal.pone.0303656 (PMC11335124; doi:10.1371/journal.pone.0303656)
Supplement: Attachment — Submitted filename: Reply to reviewers.pdf [file pone.0303656.s004.pdf]

Centre de Biologie pour la Gestion des Populations - CBGP
Campus International de Baillarguet
755 avenue du Campus Agropolis - CS 30016
34988 Montferrier-sur-Lez - France

Reply to reviewer #2
The authors have addressed most of my comments. However, there is still a bit of work to do, at least in my opinion.

L280: A gaussian distribution is bound by -inf to +inf for continuous variables, and therefore doesn't suit any of the investigated response variable. I understand this sounds trivial, and will probably not affect the results very much. It is however, a flawed assumption to choose the gaussian as default in mixed modelling. You can refer to books such as Zuur et al. 2009 for more appropriate distribution families for the models you are running.

We prefer to maintain the Gaussian distribution as a commonly-used distribution with these types of variables. In addition, the variables were standardised to mean and standard deviation equal to zero and one, respectively, which renders them in between *-inf to +inf.*

It is also my opinion that p-values poorly reflect the range of effect size from the models. For instance, we could have a scenario in which other researchers would use the effect size you present for a meta-analysis. Without a CI range, it becomes impossible to fully understand the potential range for errors, as p-values only reflects if said range crosses the 0 line or not. As such, I would strongly recommend the authors to add the CI in their results, and keep the p-values if they want to do so.

We agree with the reviewer and we have added CIs.

Minor comments:
L244: it's 'kilometers', not 'quilometers'

Corrected

Aside from that, I'm quite happy with the manuscript in its present form, and congratulate the authors for a thorough work in addressing my comments!

Bon travail!

Antoine Filion, PhD

Reply to reviewer #3
This paper is described parasitoid wasp diversity in agricultural abandonment and semi-natural habitats and provide insight to relative influence of different habitats for them selecting one wasp family as a sample organism.

Comment 1:
"In this study (1) we hypothesise that parasitoid species richness, evenness, total abundance and beta diversity will relate positively with the time since agriculture was abandoned, and with the proportion of semi-natural habitats at the landscape level." Here, could you please explain what is meant by "with the proportion of semi-natural habitats at the landscape

Centre de Biologie pour la Gestion des Populations

Centre de Biologie pour la Gestion des Populations - CBGP
Campus International de Baillarguet
755 avenue du Campus Agropolis - CS 30016
34988 Montferrier-sur-Lez - France

level"?

We have added : i.e. the proportion of semi-natural habitats in buffers surrounding study sites

Comment 2:
It is very clear that authors have selected four sites in each mountain range. But it is bit unclear in the following sentence " and two sites at different stages of succession (i.e. ten and twenty years from agricultural abandonment)". "In each mountain range, we selected four sites along a gradient of time since agriculture was abandoned, from a wild protected area to a managed cropland, and two sites at different stages of succession (i.e. ten and twenty years from agricultural abandonment)"

Corrected

Comment 3:
It would be better that scientific name of the species followed by author & year (year they described the species) eg: Mesostenus albinotatus Gravenhorst, YEAR

Done

Comment 4:
Appendix 1: It would be convenient for readers if habitats are classified as cropland, wild etc.. rather than listing their names.

This information is already included in Table 1 in the main text.
